# When Severe Hypothyroidism Mimics Neuromuscular Diseases: A Case Supporting Outpatient Management in Contemporary Endocrinology

**DOI:** 10.7759/cureus.91097

**Published:** 2025-08-27

**Authors:** Matteo Acanfora, Barbara Presciuttini, Dario Benazzi, Anna Pulcina, Mauro Pagani

**Affiliations:** 1 Institute of Endocrine and Metabolic Sciences, Istituto di Ricovero e Cura a Carattere Scientifico (IRCCS) Ospedale San Raffaele, Milan, ITA; 2 Endocrinology Unit, Vita-Salute San Raffaele University, Istituto di Ricovero e Cura a Carattere Scientifico (IRCCS) Ospedale San Raffaele, Milan, ITA; 3 Endocrinology Unit, Azienda Socio Sanitaria Territoriale di Mantova, Mantua, ITA; 4 Department of Emergency Medicine, Carlo Poma Hospital, Mantua, ITA; 5 Department of Endocrinology, Carlo Poma Hospital, Mantua, ITA; 6 Department of Internal Medicine, Carlo Poma Hospital, Mantua, ITA

**Keywords:** hypothyroidism, myopathy, neuromuscular disorders, outpatient management, subacute neurological disorders, sustainability of healthcare system

## Abstract

Severe hypothyroidism may present with symptoms similar to those of primary neuromuscular or acute neurological disorders, resulting in diagnostic delays and potentially unnecessary hospitalizations. A 40-year-old man presented to the emergency department with postural instability, diplopia, myalgia, and elevated creatine kinase, suggestive of a neuromuscular disorder. Laboratory test results revealed thyrotropin (TSH) levels over 170 μU/mL and suppressed free thyroxine (fT4). Despite severe biochemical parameters, clinical stability allowed for outpatient management with levothyroxine administration and endocrinological follow-up. The case mimicked inflammatory myopathy or brainstem stroke, but the absence of ‘red flags’ allowed for safe outpatient treatment. Literature confirms that hypothyroidism can present with neurologic signs and that structured outpatient pathways are effective. This report emphasizes the importance of recognizing endocrine causes in neuromuscular presentations and advocates for outpatient management models in stable hypothyroid patients.

## Introduction

The increasing demand for hospital resources compels healthcare systems, such as Italy’s National Healthcare System, Servizio Sanitario Nazionale (SSN), to reevaluate the need for hospital admissions, particularly for subacute and chronic conditions [1]. Within this context, endocrinology emerges as a discipline well-suited to outpatient care models. Although traditional management of severe hypothyroidism favors inpatient admission due to risks such as myxedema coma, there is growing evidence that many cases, despite pronounced biochemical abnormalities, do not present with clinical instability and can be safely managed in outpatient settings, avoiding an economic burden on the healthcare system [2,3].

This article was previously posted to the Zenodo preprint server on July 23, 2025.

## Case presentation

A 40-year-old man presented to the emergency department (ED) with a subacute onset of postural instability, diplopia, fatigue, and diffuse myalgia. He appeared weak and slow in both movement and speech. Physical examination revealed periocular edema, a heart rate of 60 beats per minute (bpm), and arterial blood pressure of 110/70 mmHg. There was no hepatosplenomegaly, and no focal limb weakness was observed. Neurological examination was otherwise unremarkable. 

Laboratory results showed a markedly elevated thyrotropin (TSH) level of 172 μU/mL (upper limit of normal (ULN) < 4.2), suppressed free thyroxine (fT4), 0.04 ng/dL (lower limit of normal (LLN) > 0.90), and elevated levels of creatine kinase (CK), 4588 U/L (ULN < 200) and creatinine (1.48 mg/dL, ULN < 1.2), with an estimated glomerular filtration rate (eGFR) of 59.2 mL/minute/1.73m² (using the Chronic Kidney Disease Epidemiology Collaboration (CKD-EPI) equation). Relevant laboratory data are presented in Table 1. Despite these concerning biochemical markers, the patient was alert, hemodynamically stable, and showed no signs of respiratory or cardiac compromise. Nevertheless, admission to the internal medicine department was requested by the ED staff. 

**Table 1:** Summary of relevant laboratory data TSH: thyrotropin; fT4: free levothyroxine; CK: creatine kinase; eGFR: estimated glomerular filtration rate

Parameter	Patient Value	Reference Range
TSH	172 μU/mL	0.4 – 4.2 μU/mL
fT4	0.04 ng/dL	0.90 – 1.70 ng/dL
CK	4588 U/L	30 – 200 U/L
Creatinine	1.48 mg/dL	0.6 – 1.2 mg/dL
eGFR	59.2 mL/min/1.73m²	> 90 mL/min/1.73m²
fT4 (Day 4)	0.15 ng/dL	0.90 – 1.70 ng/dL
fT4 (Day 20)	0.73 ng/dL	0.90 – 1.70 ng/dL
TSH (Day 20)	64 μU/mL	0.4 – 4.2 μU/mL

**Figure 1:**
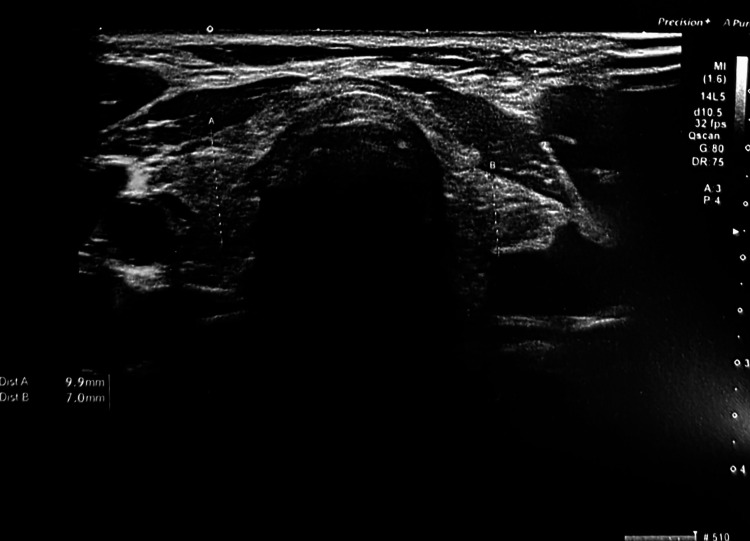
Thyroid ultrasound (anteroposterior view) The thyroid gland is hypotrophic, slightly hyperechoic, and with heterogeneous echotexture.

After consultation with the endocrinology service, a diagnosis of severe primary hypothyroidism was established. Oral levothyroxine was initiated at a dose of 50 μg/day, and the patient was discharged home with careful follow-up in the endocrinology outpatient clinic. Anti-thyroid peroxidase antibodies were elevated, supporting a diagnosis of chronic autoimmune thyroiditis. Thyroid ultrasound showed a small, slightly hyperechoic gland (Figure 1). A neurological assessment excluded central nervous system involvement.

On day four, TSH remained elevated while fT4 showed slight improvement (0.15 ng/dL). The dose of levothyroxine was increased to 75 μg/day (Table 1). By day nine, further biochemical and clinical improvement was noted. On day 20, with a serum TSH concentration of 64 μU/mL (fT4 0.73 ng/dL), marked symptomatic relief and the absence of palpitations were noted, and the dose was titrated to 100 μg/day.

## Discussion

This case highlights the variety of presentations of hypothyroidism and its ability to mimic both neuromuscular disorders and acute neurological syndromes. The constellation of symptoms, such as diplopia, gait imbalance, and elevated CK, initially suggested differential diagnoses including inflammatory myopathies or even brainstem ischemia [4,5]. The diagnostic complexity is further compounded by non-specific findings such as fatigue and mental slowing, often attributed to psychiatric or systemic illnesses. Hypothyroidism can present with polyneuropathy, entrapment syndromes, and even cerebellar dysfunction, all of which contribute to diagnostic delay [6-9]. Nevertheless, the absence of clinical red flags enabled a management strategy focused on outpatient care. 

The final diagnosis was chronic autoimmune (Hashimoto’s) thyroiditis with severe hypothyroidism, myocytolysis, and initial acute renal failure. This case illustrates that, with careful monitoring and in the absence of clinical red flags, even severe biochemical hypothyroidism can be managed safely in the outpatient setting.

Clinical stability, specialist access, and timely follow-up proved decisive in avoiding hospitalization, resulting in a better use of public healthcare system resources. 

## Conclusions

Severe hypothyroidism presenting with neuromuscular symptoms can mimic primary neurological or muscular disorders, deceptively resembling neurological emergencies. This case highlights that biochemical severity alone does not mandate hospitalization in the absence of hemodynamic or neurocognitive instability. The successful outpatient management underscores the value of integrated care between emergency medicine and endocrinology, improving diagnostic accuracy and resource stewardship. Such an approach supports clinically sound and economically sustainable healthcare models.
